# Repeated applications of high potassium elicit long-term changes in a motor circuit from the crab, *Cancer borealis*

**DOI:** 10.1016/j.isci.2022.104919

**Published:** 2022-08-11

**Authors:** Mara C.P. Rue, Leandro M. Alonso, Eve Marder

**Affiliations:** 1 Biology Department and Volen Center, Brandeis University, Waltham, MA 02454, USA

**Keywords:** Ethology, Molecular physiology, Neuroscience

## Abstract

We examined the effects of altered extracellular potassium concentration on the output of the well-studied pyloric circuit in the crab, *Cancer borealis*. Pyloric neurons initially become quiescent, then recover spiking and bursting activity in high potassium saline (2.5x[K^+^]). These changes in circuit robustness are maintained after the perturbation is removed; pyloric neurons are more robust to subsequent potassium perturbations even after several hours of wash in control saline. Despite this long-term “memory” of the stimulus history, we found no differences in neuronal activity in control saline. The circuit’s adaptation is erased by both low potassium saline (0.4x[K^+^]) and direct hyperpolarizing current. Initial sensitivity of PD neurons to high potassium saline also varies seasonally, indicating that changes in robustness may reflect natural changes in circuit states. Thus, perturbation, followed by recovery of normal activity, can hide cryptic changes in neuronal properties that are only revealed by subsequent challenges.

## Highlights

• Pyloric neurons adapt their activity in sustained high [K^+^] saline
• Repeated applications of high [K^+^] are less disruptive, even after long washes
• “Memory” of prior high [K^+^] treatment does not alter activity in control saline
• The robustness of the pyloric circuit to high [K^+^] fluctuates seasonally

## Graphical abstract

**Figure:**
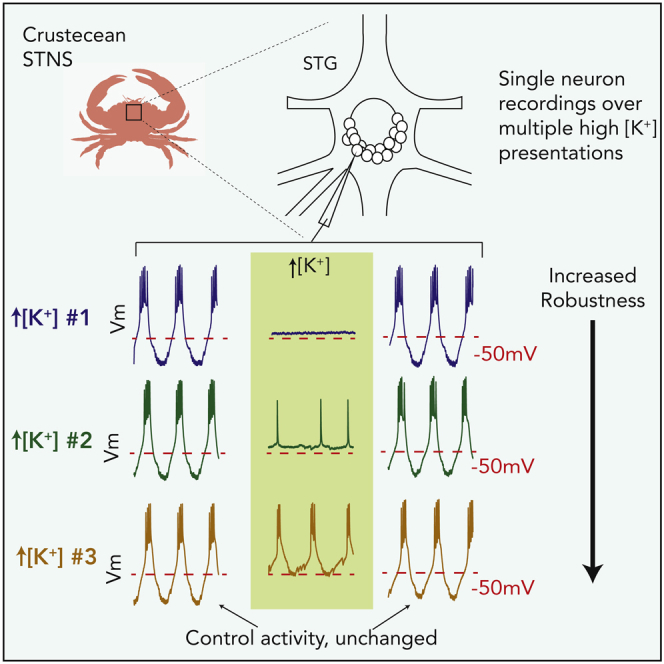

## Introduction

Neuronal circuits must adapt and survive in the face of perturbations from internal and external environments. Maintaining the appropriate ionic composition of the extracellular milieu is critical for normal physiological function, and the potassium gradient is particularly important for the maintenance of resting membrane potential and normal activity levels. It is therefore unsurprising that altered potassium concentrations occur in a wide array of conditions including heart disease, kidney failure, thermal stress, tissue damage, epilepsy, traumatic brain injury, and stroke (Arnold et al., 2014; Baylor and Nicholls, 1969; Chauvette et al., 2016; Katayama et al., 1990; Morrison et al., 2011; Rodgers et al., 2007; Seeburg and Sheng, 2008).

In addition to these pathological states, altered extracellular potassium levels are routinely used by researchers as a physiologically relevant depolarizing stimulus to increase neuronal activity or as a proxy for excitatory inputs (Ballerini et al., 1999; Ruangkittisakul et al., 2011; Rybak et al., 2014; Sharma et al., 2015). Nonetheless, many studies employing high potassium do not record the physiological response of neurons. Those that do record physiologically often observe long-term, chronic changes of populations of neurons over days to weeks (Grubb and Burrone, 2010; O’Leary et al., 2010; Rannals and Kapur, 2011). But changing extracellular potassium concentration will immediately affect neuronal membrane potentials and thus may activate rapid adaptation in addition to long-term changes in neuronal activity. In a previous study on the crustacean stomatogastric nervous ganglion (STG), we showed that the pyloric rhythm first becomes quiescent and then recovers function after many minutes in 2.5 times the physiological concentrations of K^+^ (He et al., 2020). The adaptation observed by He et al. (2020) was due to a change in the intrinsic properties of pyloric neurons and did not rely on a change in synaptic efficacy. This raised the question of whether any trace of this adaptation persisted after the preparation was returned to control saline.

Activity-dependent adaptation can occur over distinct timescales. The shortest activity-dependent adaptation processes, such as spike frequency adaptation, emerge directly because of the biophysical properties of channels and can occur on the millisecond timescale. Over longer time frames, activity-dependent homeostatic mechanisms actively regulate ion channel expression and synaptic weights to maintain stable function in the face of extended physiological perturbation (Brickley et al., 2001; Desai, 2003; Golowasch et al., 1999; Hengen et al., 2013; Marder and Goaillard, 2006; Mease et al., 2013; O'Leary and Marder, 2014; Temporal et al., 2014; Turrigiano, 2012; Turrigiano et al., 1998; Turrigiano and Nelson, 2004). These homeostatic processes are commonly thought to act over hours to days and require protein synthesis. However, feedback mechanisms can also drive more rapid adaptation over intermediate timescales on the order of minutes. For instance, phosphorylation or dephosphorylation of ion channels (Capera et al., 2019; Misonou et al., 2004; Park et al., 2006) or rapid insertion of ion channels (Frank et al., 2006) mediated by second-messenger pathways can occur over seconds to many minutes. These rapid adaptation processes are well studied in the context of learning and memory, particularly at the synaptic level (Ataman et al., 2008; Fernández-Fernández and Lamas, 2021; Li et al., 2004; Matzel et al., 1996). However, it is not clear how neurons and neuronal circuits balance rapid changes in intrinsic properties with stable baseline activity.

The crustacean STG is an excellent system with which to study underlying network dynamics and mechanisms of circuit robustness by recording from well-studied identified neurons (Haddad and Marder, 2018; Haley et al., 2018; He et al., 2020; O'Leary and Marder, 2016; Ratliff et al., 2021). The physiological output of each neuron within the STG is relatively stereotyped, allowing the determination of whether a given pattern of activity is normal. This system therefore provides an opportunity to study how a neural circuit can achieve stable adaptation to global perturbation while maintaining its characteristic physiological function (Prinz, 2014). Taking advantage of this tractable and well-defined system, we investigated the response of neurons to repeated high potassium applications and describe a case of intermediate-term (minutes) adaptation to a global perturbation that is bidirectional and retained over long time periods (hours). These studies demonstrate that rapid adaptation can lead to cryptic changes in circuit state that become visible only in response to a subsequent environmental challenge.

## Results

### Pyloric neurons rapidly adapt to elevated potassium concentrations

**Figure 1:**
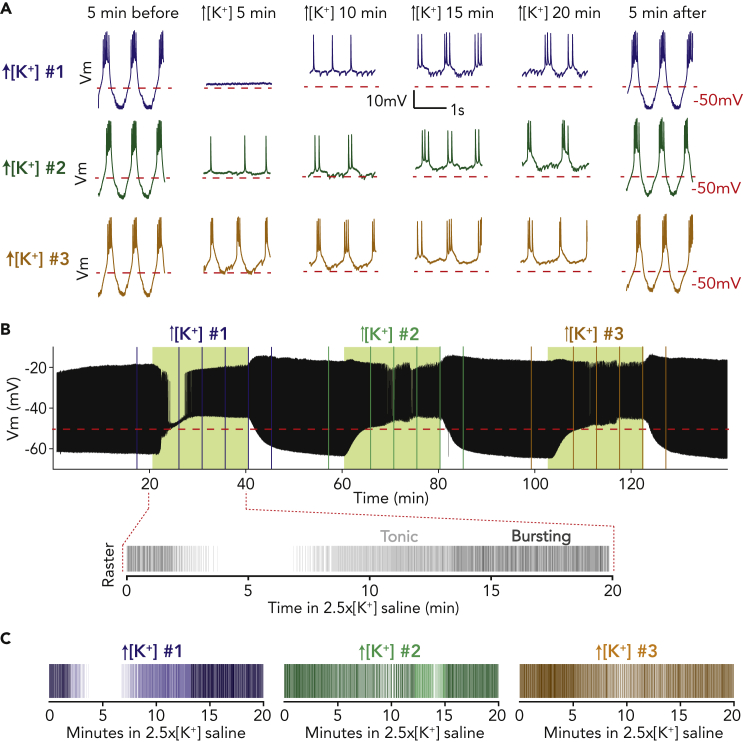
PD neurons adapt to elevated potassium concentrations and have increased robustness to repeated exposures (A) Two-second segments of the PD neuron’s activity in all three high [K^+^] applications in control physiological saline (left), and 5, 10, 15, and 20 min into each application of 2.5x[K^+^] saline and during wash (right). (B) Voltage trace for the same PD neuron over the entire experiment. Green shaded boxes indicate time of 2.5x[K^+^] saline superfusion. Below this trace is shown a raster plot of spiking activity for the entire first application of 2.5x[K^+^] saline, with bursting activity plotted in a darker shade and tonic firing plotted in a lighter shade. (C) Raster plots of spiking activity in 2.5x[K^+^] saline for all three exposures. For all rasters, bursting activity is plotted in a darker shade and tonic firing in a lighter shade.

The pyloric central pattern generator within the STG drives filtering of food particles through the foregut *in vivo* (Harris-Warrick et al., 1992). Pyloric activity *in vivo* persists *in vitro* (Rezer and Moulins, 1983; Soofi et al., 2014) and can be monitored using a combination of intracellular and extracellular recordings. The pyloric network is driven by the electrically coupled anterior burster (AB) and two PD neurons, which together form a pacemaker kernel. In this study we used the regular bursting activity of the PD neurons as a proxy for robustness of the pyloric circuit (Figure 1A, 5 min before and 5 min after traces). For all preparations, the STNS was dissected intact (including upstream modulatory commissural and esophageal ganglia) from the stomach of the crab, *Cancer borealis*, and pinned in a dish, allowing us to change the composition of the continuously superfused saline.

We previously demonstrated that pyloric neurons depolarize and temporarily become silent in high potassium saline, with the recovery of spiking activity occurring through a change in cell-intrinsic excitability (He et al., 2020). Figure 1 illustrates the typical response of a PD neuron to high potassium saline. When PD neurons are first exposed to 2.5 times the physiological concentration of extracellular potassium (2.5x[K^+^] saline), the neuron depolarizes and becomes quiescent. Subsequently, it recovers spiking and later bursting activity over 20 min in elevated extracellular potassium (Figure 1A, blue traces). This change in activity can be visualized in the raw voltage traces (Figure 1A, top) and simple raster plots where a line is plotted for each action potential in the respective PD neuron (Figure 1B, bottom). The full trace in Figure 1B shows the effects of three 20-min 2.5x[K^+^] saline exposures interspersed with 20-min washes in physiological (control) saline (Figure 1A, green and orange example traces, Figure 1B). Repeated exposure to elevated extracellular potassium resulted in shorter or diminished periods of quiescence and more robust PD neuron spiking activity compared with the initial application (Figure 1C).

**Figure 2:**
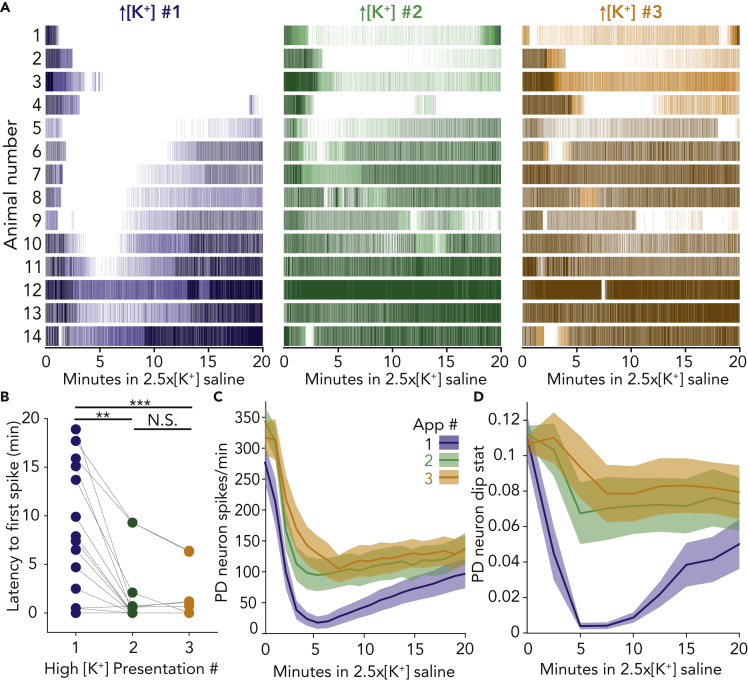
PD neurons respond more robustly to repeated applications of high potassium saline, despite no changes in control activity (A) Raster plots of spiking activity in 2.5x[K^+^] saline for fourteen PD neurons exposed to three repeated exposures. For all plots, bursting activity is plotted in a darker shade and tonic firing in a lighter shade. (B) Latency to recovery of the first action potential for each PD neuron across all high [K^+^] applications. The time of silence in the first application is significantly higher than the second (∗∗p = 0.0022) and third applications (∗∗∗p = 0.0005). (C) Average PD spikes per minute for all three applications are plotted in the dark line with ±SEM lighter shaded regions around them. (D) Average PD dip value for all three applications is plotted in the dark line with ±SEM lighter shaded regions around them.

In data from 14 animals, PD neurons exhibited more spiking and bursting behavior in high [K^+^] applications #2 and #3 compared with the first application (Figure 2A). The increase in robustness to subsequent challenges can be quantified in several ways. First, the crash time, or latency to first spike, decreases across application number for each PD neuron, and for all animals (Figure 2B, Paired Wilcoxon tests with Bonferroni correction) application 1 differs from applications 2 and 3 (p = 0.0022 and 0.0005, respectively). Application 2 does not differ from application 3 (p = 0.79). Second, the total number of spikes per minute fired by PD neurons in high potassium saline is higher in the second and third applications compared with the first (Figure 2C, Friedman’s test, Q(2) = 23.57, multiple comparisons with Bonferroni correction). The number of spikes during the first application differs from second and third for 4–13 min after beginning of application (p < 0.0025 for all).

Finally, under normal physiological conditions, pyloric neurons produce bursts of action potentials, which are necessary to drive rhythmic contractions of muscles within the stomach of the crab (Morris and Hooper, 1997). Therefore, we also characterized the “burstiness” of pyloric neurons during exposure to high potassium saline using Hartigan’s dip statistic, in which higher numbers indicate more burst-like activity (Hartigan and Hartigan, 1985). For all PD neurons, the dip statistic was higher throughout the second and third high potassium applications compared with the first (Figure 2D, Friedman’s test, Q(2) = 16.87, multiple comparisons with Bonferroni correction; 2-min bins). The dip value during the first application differs from second and third for 6–12 min after beginning of application (p < 0.005 for all). Overall, the reduced latency to spike, higher number of spikes, and “burstiness” of PD neurons in high potassium saline upon repeated applications indicate that the response of the pyloric circuit can be altered by a single exposure to high potassium and that these changes are maintained after 20-min washes in control saline.

### Pyloric activity in control saline is unchanged following potassium perturbation

**Figure 3:**
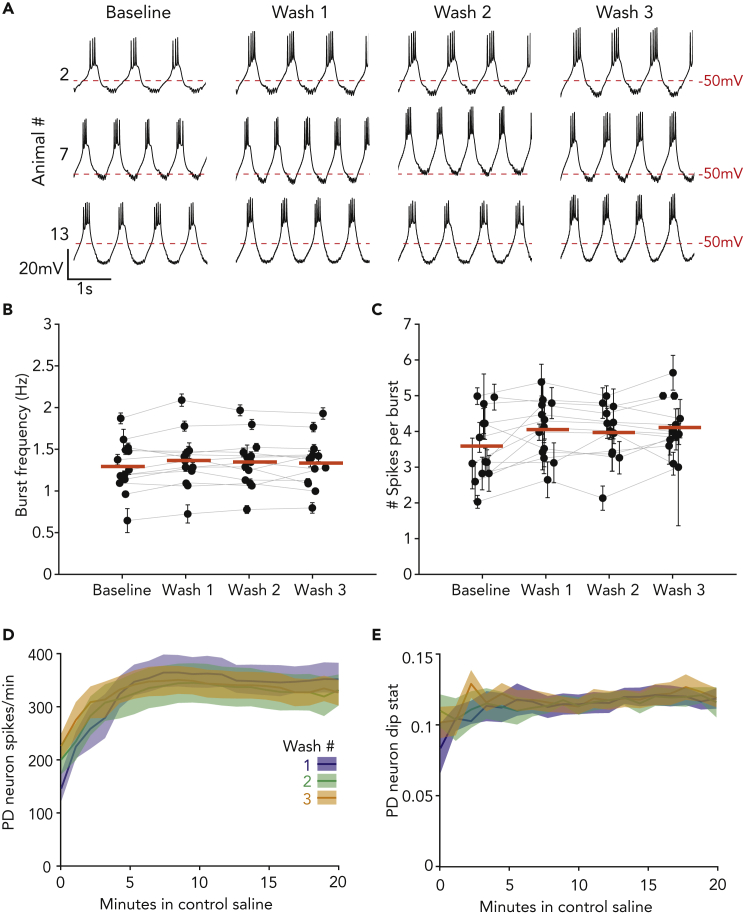
Control activity and recovery from high potassium saline are unchanged by previous exposure to 2.5x[K^+^] saline (A) Three-second segments of PD neuron activity for three animals (#2, 7, and 13, same animals as shown in Figure 2) 10 min after exposure to each high potassium saline perturbation. (B and C) Average burst frequency (B) and number of spikes per burst (C) for each neuron, and across all neurons, in control saline is unchanged after exposure to high potassium saline. For (B) and (C), the median value is represented by the thick red line, and each preparation’s variance is represented in error bars ±SEM. (D and E) Rebound of spiking activity (D) and PD dip value (E) plotted by wash number. Averages for spikes per minute and dip statistic are plotted in the dark line, with ±SEM lighter shaded regions around them.

Given that pyloric neurons rapidly adapt to the high potassium perturbation, we might expect that this change in robustness would be reflected in the neurons’ overall activity level after the perturbation was removed. To see if this were the case, we directly compared the bursting activity of each PD neuron in control saline and after each high potassium application (Figure 3A). In the example traces seen in Figure 3A, although each PD neuron (e.g., animals #2, 7, and 13) responded differently to the high potassium saline applications, the activity in control saline was unchanged. For all PD neurons, we analyzed the bursting activity in the last 10 min of baseline and washes #1–3. The burst frequency of PD neurons was unchanged in control saline regardless of the wash number (Figure 3B, Friedman’s test, Q(3) = 2.45, p = 0.46). Similarly, there was no change in the average number of spikes per burst (Figure 3C, Friedman’s test Q(3) = 4.66, p = 0.17).

We characterized the recovery trajectory of PD neurons upon return to control saline to determine if the rate of recovery was altered by application number. When high potassium saline was washed out, the number of PD spikes per minute (Figure 3D) and the bursting activity (Figure 3E) returned to baseline levels within 5 min. The rates of recovery for spikes per minute and dip statistic were not affected by the number of high potassium applications (Figures 3D and 3E, Friedman’s test, all time points N.S.). In summary, although PD neurons show robust adaptation to high potassium saline, we observe no differences in bursting behavior under control conditions.

### Adaptation to elevated potassium is maintained after several hours in control saline

Because the potassium applications we applied are relatively brief, one might expect PD neurons to return to their baseline sensitivity after a longer period in control saline, losing the enhanced robustness to high potassium saline over time.

**Figure 4:**
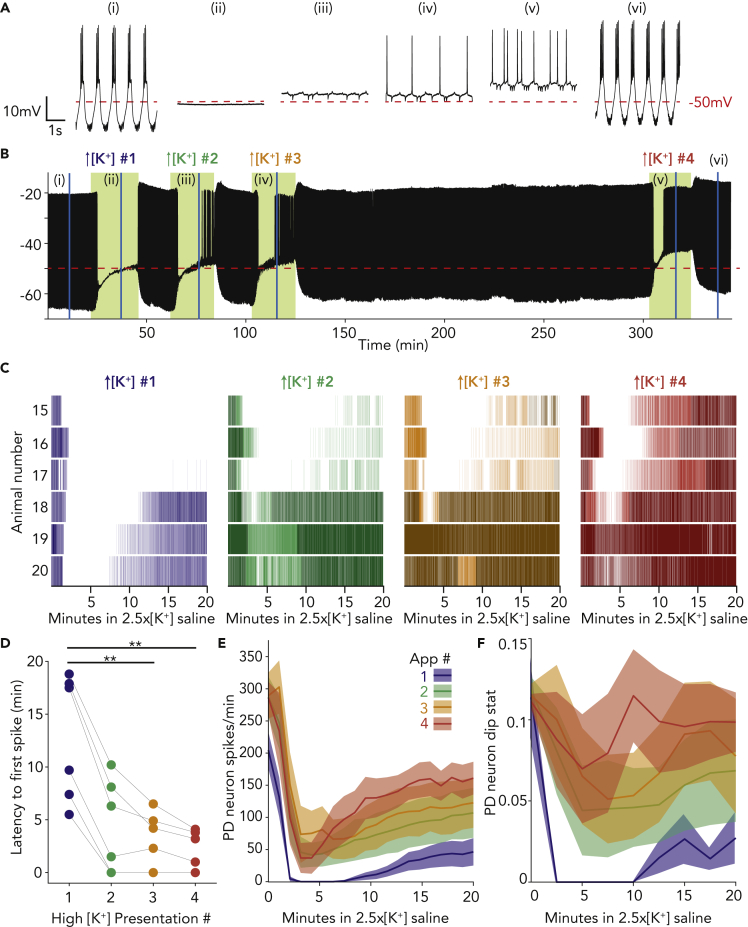
PD neurons retain adaptation to high potassium saline even after several hours of wash in control saline (A) Four-second segments of a PD neuron’s activity in control physiological saline (i), and at 15 min into the first (ii), second (iii), third (iv), and fourth (v) applications of 2.5x[K^+^] saline, and upon the final wash in control saline (vi). (B) Voltage trace for the same PD neuron over the entire experiment. Green shaded boxes indicate time of 2.5x[K^+^] saline superfusion. Below this trace is shown a raster plot of spiking activity for each of the four applications of 2.5x[K^+^] saline, with bursting activity plotted in a darker shade and tonic firing plotted in a lighter shade. (C and D) Raster plots of spiking activity in 2.5x[K^+^] saline for six PD neurons (15–20) exposed to the same four repeated exposures. For all plots, bursting activity is plotted in a darker shade and tonic firing in a lighter shade. The top raster (15) is the same animal as that shown in (A) and (B) above. (D) Latency to recovery of the first action potential for each PD neuron across all high [K^+^] applications. The time of silence in the first application is significantly higher than the third (∗∗p = 0.0043) and fourth applications (∗∗p = 0.0022). (E and F) Average PD spikes per minute for all four applications are plotted in the dark line, with ±SEM lighter shaded regions around them. (F) Average PD dip value for all three applications is plotted in the dark line, with ±SEM lighter shaded regions around the lines.

To test this, we performed additional experiments in which we applied the same three 20-min applications of 2.5x[K^+^] saline interspersed with 20-min washes in control saline. Next, we applied a 3-h wash and finally a fourth 20-min 2.5x[K^+^] saline application. Unlike in the previous set of experiments, this third wash was many times longer than the perturbations that drove changes in robustness. Again, PD neurons showed improved robustness over the first three applications of high potassium saline (example traces of activity at 15 min in 2.5x[K^+^] saline, Figure 4Aii, iii, iv). After the 3-h wash in control saline, the representative PD neuron (animal 15) shown in Figure 4A still showed improved robustness to 2.5x[K^+^] saline (Figure 4A*v*, 4B). All PD neurons in this set of six experiments retained the adaptation to high potassium saline after extended wash (Figure 4C). As in the PD neurons shown in Figure 2, the latency to first spike decreased across application number for each PD neuron and for all animals (Figure 4D, paired Wilcoxon tests with Bonferroni correction, application 1 differs from applications 3 and 4, p = 0.0043 and 0.0022, respectively). Application 1 does not differ from application 2 (p = 0.063). Note that the latency to first spike did not change between applications 3 and 4, despite the extended wash period in control saline (Figure 4D, paired Wilcoxon test with Bonferroni correction, p = 0.45). Overall, the number of spikes per minute in 2.5x[K^+^] saline increased across the first three applications and was maintained in the fourth application after the extended wash (Figure 4E, Friedman’s test Q(3) = 18.54, multiple comparisons with Bonferroni correction). The number of spikes per minute during the first application differed from second, third, and fourth for 2–14 min after the beginning of application (p < 0.0025 for all). PD neurons exhibited more bursting activity in high potassium saline in applications #2–4 compared with the first (Figure 4F, Friedman’s test Q(3) = 10.39, multiple comparisons with Bonferroni correction; 2-min bins). Dip statistic during the first application differed from second, third, and fourth for 2–12 min after beginning of application (p < 0.005 for all). In a few preparations (N = 3), we extended the interim wash period to 6 h between the third and fourth high potassium application and found that even at this longer time point PD neurons were more robust to the fourth 2.5x[K^+^] saline perturbation (median latency to first PD spike in across the four applications = 8.8, 3.6, 2.9, and 1.4 min, respectively). Thus, pyloric neurons retained an imprint of past exposures to high potassium saline, even after a wash period much longer than the perturbation itself and even though in the control saline there was no overt sign that the adaptation had taken place.

### Brief exposure to hyperpolarizing stimuli can erase adaptation

**Figure 5:**
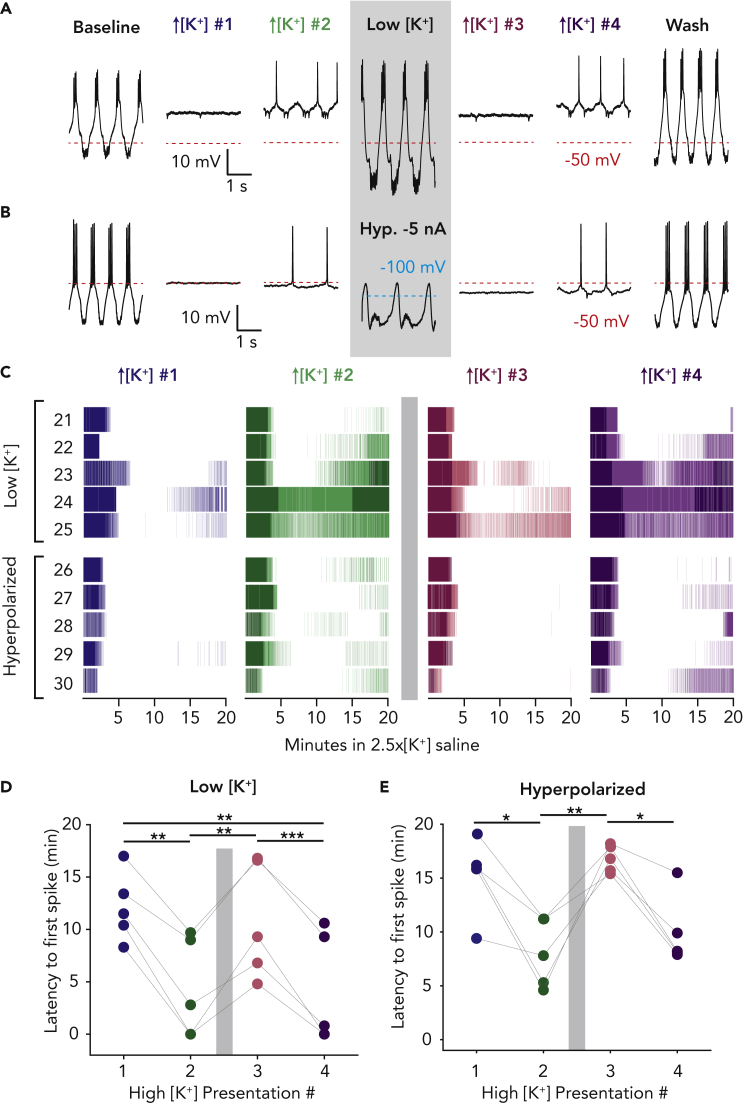
Adaptation to high potassium can be erased by a hyperpolarizing stimulus (A and B) PD neurons were exposed to two 20-min high potassium (2.5x[K^+^]) saline exposures, followed by a hyperpolarizing stimulus, then two more high potassium exposures. All perturbations were interspersed with 20-mintue washes in control saline. All activity traces are taken from 15 min into each perturbation. For all panels, gray boxes indicate time of hyperpolarizing stimulus. (A) Three-second segments of a PD neuron’s activity in all high potassium applications and the low potassium (0.4x[K^+^]) perturbation. (B) Three-second segments of another PD neuron in all high potassium applications and during direct hyperpolarization by injected current. (C) Raster plots of spiking activity in high potassium saline for nine PD neurons (21–30) exposed to the same four repeated exposures, with a hyperpolarizing stimulus between the second and third high potassium exposure. For all rasters, bursting activity is plotted in a darker shade and tonic firing in a lighter shade. Animals 21–25 were exposed to low potassium and animals 26–30 had the PD neuron directly hyperpolarized with injected current. (D) Low potassium experiments: latency to recovery of the first action potential for each PD neuron across all high [K^+^] applications. (E) Direct hyperpolarization experiments: latency to recovery of the first action potential for each PD neuron across all high [K^+^] applications. ∗p < 0.02, ∗∗p < 0.01, ∗∗∗p < 0.0001.

We next asked if we could reverse the cryptic memory by presenting an opposite stimulus to the depolarizing high potassium perturbation. We superfused two high potassium (2.5x[K^+^]) saline applications, followed by one of two kinds of hyperpolarizing perturbation, and finally two more high potassium applications. All perturbations were 20-min long and interspersed with 20-min washes in control saline. The first hyperpolarizing stimulus we tested was low potassium (0.4x[K^+^]) saline. In the representative traces shown in Figure 5A, the PD neuron exhibited a typical adaptation to high potassium saline and fired more spikes in the second high potassium presentation compared with the first. In low potassium saline, the minimum membrane potential of the PD neuron hyperpolarized by approximately 15 mV and maintained its typical bursting activity. Following the low potassium exposure, in the third high potassium perturbation, the PD neuron was less robust and responded much as it did to the first application. Finally, the pyloric circuit reacquired some robustness, and the PD neuron fired more action potentials during the fourth high potassium perturbation. Figure 5B shows the response of another PD neuron to the same experimental protocol, except instead of low potassium saline the PD neuron was hyperpolarized with direct current until it became silent and held at that membrane potential for 20 min. Again, the hyperpolarizing stimulus reduced the robustness of the PD neuron to the next presentation of high potassium saline.

Across all animals exposed to low potassium or hyperpolarizing current, the PD neurons were less robust to the subsequent high potassium perturbation (Figure 5C). This change in robustness cannot be explained by extended time between high potassium perturbations, as we showed in Figure 4 that PD neurons retain adaptation to high potassium saline in wash for at least 3 h. For preparations exposed to low potassium (Figure 5D) and hyperpolarizing current (Figure 5E), there was an increase in time to first spike between high potassium applications #2 and #3 (Paired Wilcoxon rank-sum test with Bonferroni correction, p = 0.0025 and 0.0079, respectively). This result stands in contrast to PD neurons exposed to high potassium saline in three or four repeated exposures, in which the latency to first spike was smaller with each presentation of high potassium saline (Figure 2, Figure 4B and 4D).

The number of spikes per minute in 2.5x[K^+^] saline increased across the first two applications and was diminished in the third application after either hyperpolarizing stimulus (Low [K^+^] Friedman’s test Q(3) = 12.94, Hyperpolarization, Friedman’s test Q(3) = 16.43, for all multiple comparisons with Bonferroni correction). The number of spikes per minute during the first and third applications differed from second and fourth for 5–16 min after the beginning of application (p < 0.0025 for all). The PD neurons also exhibited more bursting activity in high potassium saline in applications #2 and 4 compared with the first and third (Figure 4F, Low [K^+^] Friedman’s test Q(3) = 10.52, Hyperpolarization or low [K^+^] application, Friedman’s test Q(3) = 9.39, multiple comparisons with Bonferroni correction; 2-min bins). Dip statistic during the first application differed from second, third, and fourth for 6–12 min after beginning of application (p < 0.005 for all).

### Pyloric neuron’s robustness to the first high potassium saline presentation is seasonally variable

There is a large variability in individual PD neurons’ robustness to the first high potassium saline application. In particular, note the individual differences between latency of the first spike in PD neurons for the first application (Figure 2, Figure 4, Figure 5B, 4D, 5D, and 5E). Indeed, these large differences in individual sensitivity can lead to different initial means for latency of the first spike across experimental groups (compare Figure 2, Figure 5B with 5D and 5E). Therefore, we investigated what experimental conditions might contribute to the initial sensitivity of a PD neuron to high potassium saline.

**Figure 6:**
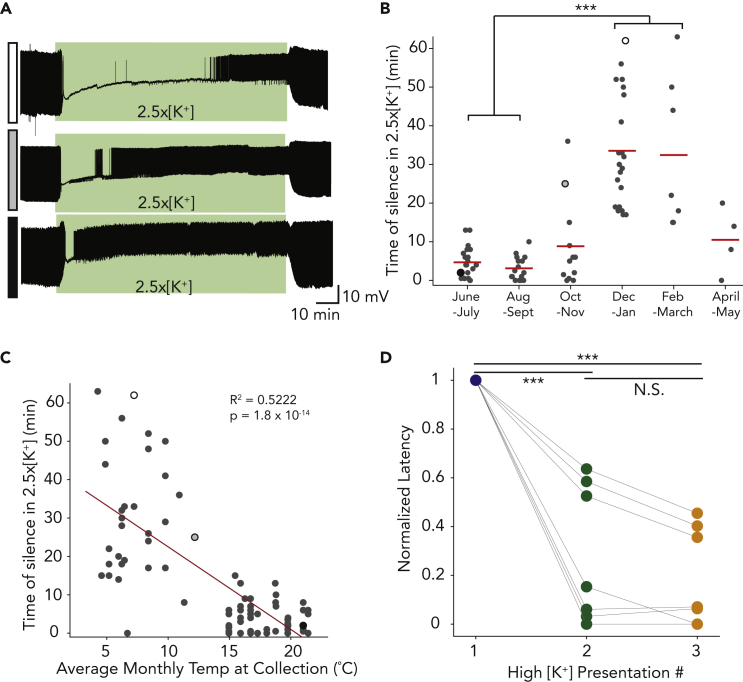
Robustness to high potassium saline varies along with the time of year and local seawater temperatures (A) Representative traces from three PD neurons with different robustness to a 90-min application of high potassium saline (green boxes). (B) Time of each PD neuron’s silence in high potassium saline grouped by month of crab collection (N = 82). Red lines indicate the mean time of silence for each two-month bin. (C) Visualization of the same data shown in (B), with each PD neuron’s time of silence in high potassium saline plotted against the average surface seawater temperature during the month the crabs were collected. Black outlined dots in (B) and (C) correspond to the traces shown in (A), pattern code in left-hand boxes. Temperature data were compiled from NOAA (https://www.ndbc.noaa.gov/station_history.php?station=44013). ∗∗∗p < 0.001. (D) Normalized latency to first spike averaged for each preparation to the latency in the first high potassium application, re-analyzed data from Figure 2. Normalized latency to recovery of the first action potential for each PD neuron across three high potassium perturbations. The time of silence in the first application is significantly higher than the second (∗∗∗p = 1.75 x 10^−6^) and third applications (∗∗∗p = 1.55 x 10^−6^).

The least robust PD neurons can be silent for 60 min (or more) in sustained 2.5x[K^+^] saline, and the most robust PD neurons exhibit brief or no silence in 2.5x[K^+^] (Figure 6A). We have done studies over more than five calendar years under the same conditions with intracellular recordings from PD neurons in 2.5x[K^+^] saline for 1 h or more. We reanalyzed and compared the time of silence in 2.5x[K^+^] saline for these 82 PD neurons from 82 animals recorded from 2016 to 2022 (recordings from Lily He, Daniel Powell, Ekaterina Morozova, Mara Rue, and Janis Li, some of which were included in He et al. (2020)). There was a striking association between the latency to first spike in high potassium and the season in which the experiment was done. The 2-month period each crab was collected has a significant effect on the PD neurons’ crash time in 2.5x[K^+^] saline (Figure 6B, Kruskal-Wallis). Crash periods during the months when the seawater temperature was lowest (Dec/Jan and Feb/Mar) were significantly different from crash periods when the seawater temperature was the highest (June/July and Aug/Sep, Kruskal-Wallis with Bonferroni correction for multiple comparisons; all p < 0.013). There was a negative correlation between each preparation’s crash time in 2.5x[K^+^] saline and the average surface seawater temperature when the crab was collected (Figure 6C, Linear Regression, R^2^ = 0.522, p = 1.8 x 10^−14^).

Given this seasonal effect on the initial sensitivity to 2.5x[K^+^] saline, we asked whether the initial PD spike latency influenced the amount or retention of adaptation to multiple high potassium applications. For the initial set of experimental animals (animals #1–14) exposed to three rapid 20-min 2.5x[K^+^] saline potassium applications, we normalized the latency to first PD neuron spike in the first application. This normalization allows us to ask whether all preparations adapt to the high potassium application regardless of the initial sensitivity. All PD neurons show a marked decrease in normalized latency to first spike from the first application to the second and the third (Figure 6D, paired Wilcoxon rank-sum test, p = 1.75 × 10^−6^ and p = 1.55 × 10^−6^, respectively); this indicates that although the response of PD neurons to high potassium saline changes seasonally, we see robust adaptation across multiple applications regardless of their initial sensitivity.

## Discussion

Nervous systems are both blessed and cursed with flexibility. To maintain stable function and respond appropriately to changes in the environment, neurons and neuronal circuits can adapt and change over timescales ranging from milliseconds to a lifetime. Therefore, there are a plethora of activity-dependent mechanisms that regulate neuronal excitability and overall circuit output that act over timescales from milliseconds to years. Rapid plasticity on the order of milliseconds to seconds, such as seen in spike frequency adaptation or synaptic facilitation, can arise from ion channel properties. These processes are critical for shaping neuronal responses and may play a role in shaping working memory, signal transduction, and many behaviors (Benda et al., 2005; Roach et al., 2016). Activity-dependent changes in excitability can also occur on timescales of several to many minutes (Golowasch et al., 1999). On these timescales, changes in effective conductance densities often occur through calcium-dependent or voltage-dependent signaling cascades, leading to phosphorylation or insertion of ion channels (Ewald et al., 1985; Frank et al., 2006; Misonou et al., 2004; Park et al., 2006).

These different activity-dependent processes, occurring on different timescales, have often been studied as separate, nonoverlapping processes. But real neurons must transition between multiple adaptation mechanisms seamlessly. In this study, we describe rapid adaptation in pyloric neurons following global depolarization by high potassium; this adaptation has a long-lasting effect on the circuit and affects the neuron’s response to future high potassium applications although the baseline activity appears unchanged in control saline. This behavior reflects a bridge between timescales of adaptation in which rapid activity-dependent adaptation to global perturbation is retained long-term. Long-lasting memory of high [K^+^] perturbation can be disrupted by subsequent hyperpolarization, suggesting that this adaptation is based on an activity- or voltage-dependent process. Activity-dependent mechanisms such as this have been well described, including phosphorylation and dephosphorylation events that can alter conductances, synaptic strength, and intrinsic properties of neurons. For instance, G-protein inwardly rectifying potassium channels (GIRK) have been shown to be bidirectionally regulated by neuronal activity patterns and contribute to the overall excitability of the neuron (Fernández-Fernández and Lamas, 2021; Lalive et al., 2014). Conductances can be relatively stable under control conditions but are modulated more strongly when the cell deviates from its normal membrane potential. This sort of mechanism could explain how pyloric neurons maintain a long-term cryptic memory after high potassium exposure that can nevertheless be erased by hyperpolarizing stimuli. Within the STG, we have found stress-related heat shock proteins are upregulated following high potassium application (unpublished data), indicating that there is a robust transcriptional response to 2.5x[K^+^] saline.

### Persistent, cryptic memory in neurons following perturbation

Theoretical and experimental evidence shows that seemingly identical activity patterns in neurons can arise from widely variable underlying parameters (Alonso and Marder, 2019; Lane et al., 2016; O'Leary and Marder, 2016; Prinz et al., 2004; Sakurai et al., 2014; Schulz et al., 2006). Therefore, although PD neuron activity appears unaltered when recorded in control saline between high potassium applications, it is likely that the balance of conductances has changed, but in a way that is cryptic, or invisible, until the next challenge. In this way, past exposure to high potassium saline acts as a prior, e.g. a past experience that will bias the outcome of a future output (Darlington et al., 2017; Verstynen and Sabes, 2011; Yang et al., 2012). An interesting implication of this study is that accumulated changes in circuit robustness due to experiences over a lifetime could lead to the very individual variability we observe between identified neurons.

### Seasonal variations in robustness

It is interesting to ask whether animals that live for many years in temperate environments maintain traces of their environmental history. Therefore, it is striking we see strong seasonal effects of sensitivity to initial high K^+^ perturbations. This analysis suggests that robustness to high potassium saline in the pyloric circuit is labile across the population.

There are several (nonmutually exclusive) possibilities for the observed seasonal difference in robustness to high potassium saline. Modulation state or intrinsic conductances of the stomatogastric nervous system may differ across the year, as fluctuations in some crustacean neurohormones are known to change on a yearly cycle (Rodriguez-Sosa et al., 1997); this could lead to summer pyloric rhythms that have intrinsic properties more likely to be robust to 2.5x[K^+^] saline. Indeed, we cannot rule out that changes in modulation state also affect the rapid adaptation of the pyloric circuit to subsequent high potassium applications. Support for the first hypothesis will require future studies with systematic measurements of pyloric neurons’ intrinsic properties and modulation state across seasons.

Pyloric neurons in the summer could also have more rapid adaptation processes that allow them to recover faster regardless of initial properties. In support of this second hypothesis, it is well established that the metabolism of crustaceans is higher in warmer environments (Childress et al., 1990; Dissanayake and Ishimatsu, 2011; Iguchi and Ikeda, 1995). Thus, it is possible that increased metabolic rate could protect against other challenges involving active feedback mechanisms.

### High potassium perturbations in experiments and medicine

The concentration of potassium both inside and outside cells is critical for proper physiological function. This study highlights the possible consequences of even brief changes in extracellular potassium in a neuronal circuit. Acute elevation of potassium concentration is often used in experiments to rapidly excite or depolarize neurons as a proxy for excitatory inputs (Ballerini et al., 1999; Rybak et al., 2014; Sharma et al., 2015). Here, we demonstrate that adaptation to elevated high potassium saline can occur rapidly and appreciably change circuit properties within minutes (He et al., 2020). Therefore, studies using high potassium saline or other depolarizing stimuli may evoke rapid changes in neuronal responses to future perturbation. Notably, adaptation acquired when neurons are stimulated with high potassium can be retained long after the perturbation has passed, even if baseline activity reverts and appears to be unchanged. This phenomenon could be salient when considering the potential long-term effects of epileptic seizures and kindling (Chauvette et al., 2016). Within a seizure locus, extracellular potassium levels rapidly increase (Fröhlich et al., 2008; Moody et al., 1974) and may elicit effects that are maintained even after activity returns to normal levels. This sort of cryptic adaptation could exacerbate or ameliorate the severity of repeated seizures in the same focus. Similarly, these dynamics have been shown to affect peripheral nerves in patients with chronic kidney disease (Arnold et al., 2014). Thus, long-term adaptation could have implications for a host of disease states involving repeated insults associated with high extracellular potassium.

### Limitations of the study

Using wild-caught study animals imposes limitations on this study. As we are unable to directly control the environment or life history of the crabs we study, any seasonal effects observed here are correlational. We cannot exclude the likely possibility that variables other than temperature also fluctuate annually and may be responsible for the patterns we observed.

Another limitation of this study is that the individual variability combined with the strong adaptation phenotype made it difficult to complete a positive control using depolarizing current to complement our hyperpolarization experiments. Therefore, at present we are unable to determine whether all depolarizations would lead to long-term adaptation or whether this phenotype is high potassium specific.

## STAR★Methods

### Key resources table

**Table:** 

REAGENT or RESOURCE	SOURCE	IDENTIFIER
**Experimental models: Organisms/strains**		

*C. borealis*	Commercial Lobster	N/A

**Chemicals, peptides, and recombinant proteins**		

NaCl	Thermo Fisher Scientific	Cat#S271-3
MgCl_2_	Sigma-Aldrich	Cat#M9272
CaCl_2_	Thermo Fisher Scientific	Cat#C-614
Maleic acid	Sigma-Aldrich	Cat#M0375
Trizma base	Sigma-Aldrich	Cat#T2788
KCl	Thermo Fisher Scientific	Cat#P330
Potassium gluconate	TCL Chemicals	Cat#0040
HEPES buffer	Sigma-Aldrich	Cat#H-7637
NaSO_4_	Thermo Fisher Scientific	Cat#S415
K_2_SO_4_	Thermo Fisher Scientific	Cat#P304

**Software and algorithms**		

MATLAB	This paper	https://github.com/marderlab

### Resource availability

#### Lead contact

Further information and requests for resources and reagents should be directed to and will be fulfilled by the Lead Contact, Dr. Eve Marder (marder@brandeis.edu).

#### Materials availability

This study did not generate new unique reagents.

### Experimental model and subject details

#### Animals

Adult male Jonah Crabs, *Cancer borealis*, (N = 115) were obtained from Commercial Lobster (Boston, MA) between December 2016 and August 2020 and maintained in artificial seawater at 10–12°C in a 12-h light/dark cycle. On average, animals were acclimated in the laboratory for one week before use.

### Method details

#### Dissections

Prior to dissection, animals were placed on ice for at least 30 min. Dissection of the crab stomatogastric nervous system (STNS) were performed in two parts, the gross and the fine dissection. Breifly, in the gross dissection the stomach was dissected from the animal. Subsequently in the fine dissection we removed the intact stomatogastric nervous system (STNS) from the stomach under a dissection microscope using fine micro-dissection tools. The STNS includes the commissural ganglia, esophageal ganglion, and STG with connecting motor nerves (Gutierrez and Grashow, 2009). The STNS was pinned in a Sylgard-coated (Dow Corning) dish and continuously superfused with 11 °C saline.

#### Solutions

Physiological (control) *Cancer borealis* saline was composed of 440 mM NaCl, 11mM KCl, 26 mM MgCl_2_, 13 mM CaCl_2_, 11 mM Trizma base, 5 mM maleic acid, pH 7.4–7.5 at 23°C (approximately 7.7–7.8 pH at 11°C). High [K^+^] saline (2.5x[K^+^], 27.5mM KCl) was prepared by adding more KCl salt to the normal saline. The additional 15.5mM of KCl added to the saline results in a 3% increase in total osmolarity of the saline solution. The pyloric rhythm is robust to changes of osmolarity of at least +/− 25%, and therefore this small change in osmolarity alone is unlikely to cause any physiological changes in pyloric neurons (unpublished data).

#### Electrophysiology

Intracellular recordings from STG somata were made in the desheathed STG with 10–30 MΩ sharp glass microelectrodes filled with either an internal solution: 10 mM MgCl_2_, 400 mM potassium gluconate, 10 mM HEPES buffer, 15 mM NaSO_4_, 20 mM NaCl (Hooper et al., 2015) or 0.6M K_2_SO_4_ with 20mM KCl. Intracellular signals were amplified with an Axoclamp 900A amplifier (Molecular Devices, San Jose). Extracellular nerve recordings were made by building wells around nerves using a mixture of Vaseline and 10% mineral oil and placing stainless-steel pin electrodes within the wells to monitor spiking activity. Extracellular nerve recordings were amplified using model 3500 extracellular amplifiers (A-M Systems). Data were acquired using a Digidata 1440 digitizer (Molecular Devices, San Jose) and pClamp data acquisition software (Molecular Devices, San Jose, version 10.5). For identification of Pyloric Dilator (PD) neurons, somatic intracellular recordings were matched to extracellular action potentials on the pyloric dilator nerve (pdn) and/or the lateral ventricular nerve (lvn).

#### Elevated [K^+^] saline application

Baseline activity of the PD neuron was recorded for 30 min in control saline. The STNS was then superfused with 2.5x[K^+^] saline for 20 min, followed by a 20-min wash in control saline. This pattern was repeated, alternating between 20 min 2.5x[K^+^] saline and physiological control saline three times. In some experiments, the preparation was then washed in physiological saline for three or six hours before a final fourth 20-min 2.5x[K^+^] saline application and a final 20-min wash. For reversal experiments, a preparation was exposed to two 20-min 2.5x[K^+^] applications followed by 20-min washes in control saline. Following the second wash, the preparation was either exposed to low (0.4x[K^+^]) saline or the PD neuron was hyperpolarized until it fired no action potentials. Finally, the preparations were exposed to two more 2.5x[K^+^] saline applications and a final 20-min wash.

### Quantification and statistical analysis

#### Data acquisition and analysis

Recordings were acquired using Clampex software (pClamp Suite by Molecular Devices, San Jose, version 10.5) and visualized and analyzed using custom MATLAB analysis scripts. These scripts were used to detect and measure voltage response amplitudes and

membrane potentials, plot raw recordings and processed data, generate raster plots, and perform some statistical analyses.

#### Statistics

Statistical analysis and plotting were carried out using MATLAB 2020b built in functions for all analyses as described above. All electrophysiology analysis scripts are available at the Marder lab GitHub (https://github.com/marderlab). Details of each statistical test can be found in the results, figures, and corresponding figure legends. For all analysis we defined a significant result as a p value less than 0.05. In analysis involving multiple comparisons we used the Bonferroni correction to determine significance. No data or subjects were excluded from final analysis. For all datasets, we first performed a test for normalcy to determine the appropriate statistical analysis.

#### Analysis of inter-spike interval distributions

To extract spike times, we used a custom spike identification and sorting software (called “crabsort”) which uses a TensorFlow based machine-learning algorithm. Crabsort is freely available at https://github.com/sg-s/crabsort and its use is described in Powell et al. (2021). Distributions of inter-spike intervals (ISIs) were calculated within 2-min bins. Hartigan’s dip test of unimodality (Hartigan and Hartigan, 1985) was used to obtain the dip statistic for each of these distributions. This dip statistic was compared to analysis in Hartigan and Hartigan (1985) to find the probability of multi-modality. The test creates a unimodal distribution function that has the smallest value deviations from the experimental distribution function. The largest of these deviations is the dip statistic. The dip statistic shows the probability of the experimental distribution function being bimodal. Larger value dips indicate that the empirical data are more likely to have multiple modes (Hartigan and Hartigan, 1985). For visualizing spiking activity in raster plots, if the dip statistic was 0.05 or higher the neuron was considered to be bursting. If the dip statistic was lower than 0.05 the neuron was considered to be tonically firing. In neurons with less than 30 action potentials per minute, there were too few spikes to calculate an accurate dip statistic and the neurons are labeled as tonically firing.

## Data Availability

• All data reported in this manuscript will be shared by the lead contact upon request. • The MATLAB analysis code used in this manuscript is available at the Marder lab GitHub (https://github.com/marderlab) upon publication. • Any additional information required to reanalyze the data reported in this paper is available from the lead contact upon request.
